# Ten simple rules for preparing a job application for an academic position

**DOI:** 10.1371/journal.pcbi.1014889

**Published:** 2026-10-27

**Authors:** Lourdes Peña-Castillo, Oscar Meruvia-Pastor

**Affiliations:** 1 Department of Computer Science, Memorial University of Newfoundland, St. John’s, Newfoundland, Canada; 2 Department of Biology, Memorial University of Newfoundland, St. John’s, Newfoundland, Canada; Carnegie Mellon University, UNITED STATES OF AMERICA

## Introduction

In many academic institutions, faculty hiring committees (also called search or recruitment committees, or assessment boards) are formed to evaluate candidates, conduct interviews, and recommend candidates for employment. A general academic hiring process consists of the following stages: 1) call for applications, 2) eligibility screening, 3) pre-/screening interviews, 4) shortlisting, 5) interviews, 6) post-interview verifications, and 7) offer (see [1] for advice on navigating this process). However, the exact process varies between institutions. For example, some institutions might not carry out pre-interviews or screening interviews, which are short (around 30 min) online meetings with selected candidates. Another common variation is to split the submission of application materials: Applicants initially submit an application form, cover letter, and curriculum vitae (CV), and after an initial screening, candidates advancing in the hiring process will be asked to provide additional material. When and how reference letters must be submitted is also a common source of variation, with some institutions requesting the contact information of the referees at stage 1 and asking directly for their letters at some point between stages 3 and 6 of the process, while other institutions request that applicants arrange for their referees to submit their letter to the institution at stage 1 or later.

In addition, there is a lack of consensus on the required documents. To illustrate this, we looked at 15 faculty position ads from a diverse group of institutions, including research-intensive and teaching-focused universities, from five continents (10 countries), and a variety of disciplines (e.g., Science, Engineering, Humanities, and Law). We restricted our informal survey to ads in English (14) or Spanish (1). Table 1 lists the documents requested in at least two job ads in our survey. The only document universally required is the CV. 80% of the ads we studied required a cover letter, 67% of the ads required at least one research-related material, and 60% asked for at least one teaching-related material. Advising on the writing of each type of document is outside the scope of these ten rules; we refer the reader to the references provided in Table 1 for articles on how to write some of the requested documents.

**Table 1:** Documents required in application packages. Documents are listed from most common to least common across 15 faculty position ads. Documents required by only one institution are not listed. References provide advice on writing each specific document type.

Document type	Required	
	#	%
CV [2,3]	15/15	100
Cover letter (also called letter of interest or motivation letter) [4]	12/15	80
References	11/15	73
Application form	9/15	60
One or more research-related materials such as:	10/15	67
- Sample contributions (e.g., publications, portfolio, writing samples, and achievement descriptions)	7/15	47
- Research statement [5,6]	6/15	40
- Publication list	4/15	27
- Research plan [7]	2/15	13
One or more teaching-related materials such as:	9/15	60
- Teaching philosophy [8]	5/15	33
- Teaching/mentoring statement [9,10]	4/15	27
- Evidence of teaching effectiveness (e.g., teaching evaluations) [11,12]	4/15	27
- Sample teaching materials	3/15	20
- Teaching interests	2/15	13
Equity, diversity, inclusion, and accessibility (EDIA) statement [13,14]	2/15	13

In the last 6 years, we have cumulatively participated (as committee members or chairs) in a dozen faculty searches and in two searches for higher education administrators at Memorial University of Newfoundland, a comprehensive Canadian public university with roughly 18,000 students. During these hiring processes, we have collectively reviewed hundreds of job applications. These ten rules are based on our experiences and discussions on these search committees during stage 2 of the process (i.e., eligibility screening). In this stage, candidates are assessed against an established rubric, based on information provided in the application documents, to identify those candidates who meet the criteria for further assessment. The vast majority of applicants will be filtered out at this step (for example, at our university only 10–15 applicants will move on to the next stage). Thus, making sure that your application documents set you apart from the other candidates by clearly communicating why the search committee should move forward with your application is critical. For example, when applying to a research-oriented institution, it would be essential to communicate a compelling vision of your future research plans. Getting an academic job usually takes multiple attempts [15]. However, candidates can increase their chances of progressing through the hiring process by handing in job application documents that effectively show that they are a good fit and that their qualifications meet the selection criteria. These rules are meant to help first-time applicants to put together a good academic job application that will increase their chances of advancing past the eligibility screening. The rules outlined here complement the previously published *ten simple rules* on writing a cover letter [4] and on giving an academic job talk [16], and are loosely given in chronological order as candidates would apply them.

## Rule 1: Study the job posting

Read the job advertisement carefully. First, many job posts contain clues about the criteria that will be used to evaluate the candidates; if you identify these criteria, then you can directly address them in your job application documents. Some job ads list these criteria under the heading “selection criteria”, “requirements”, or “qualifications”. Some ads will separate the requirements into *essential* and *preferred*. Both types of requirements are important and should be addressed in your application. Second, you need to decide whether the position is right for you and whether you meet most of the requirements. If the mismatch between your qualifications and the ad is clear, it might not be worth the time and effort to submit a job application for this specific position. We suggest making a list of all the criteria (essential and preferred) that you identified in the first step and checkmarking those that you (partially) meet. Try not to discourage yourself easily. Your chances might be better than you think. If you meet roughly 50% or more of all the criteria and at least 30% of the essential criteria, then go for it! Alternatively, you could seek advice regarding your fit for the position from a senior academic in your research area (e.g., a current/past academic supervisor, mentor or close collaborator). Third, note the documents requested and the submission instructions. Elements required for a job application vary (see Table 1), but in most cases you will need to provide a cover letter, CV, references or letters of recommendation, and samples of previous research and/or publications [17]. Pay attention to details: Are they asking for a research statement or a research plan? Do you need to include a document describing your teaching philosophy? Are there page, word, or character limits? Are specific formats requested? Being meticulous in putting your application package together always helps. Many ads indicate whom to contact with questions regarding the position or the process. In general, enquiries are informal and should be specific. When making an enquiry, consider that the contact person might not be part of the hiring committee, and may not decide on a particular applicant’s eligibility or potential. Wait at least five business days before prompting the contact person again. If you do not receive a timely and professional response, consider this when assessing whether that institution looks like a promising place to work. If an ad does not provide contact information, our advice is to refrain from reaching out to someone whose involvement in the hiring process you are unsure of.

## Rule 2: Do your homework

Learn about the hiring group (institution, faculty, and/or department/unit) and make sure your application documents reflect that you have looked at them and thought about how you fit in. For example, look at some of their research and identify potential gaps and collaborators, potentially including other faculties at the same institution; look at courses and programs being offered and identify which ones you can teach and how you could enrich their curriculum; and read their vision statement or strategic goals and consider how you can contribute to achieving them. Consider contacting a faculty member recently hired or working in a related area, as they might provide insights about the hiring group and university. Alternatively, if someone from your network is at the university posting the ad, feel free to reach out to them with questions regarding the university, position and/or hiring unit. We suggest limiting your questions to the type that could help you produce a better application package and avoiding giving any impression that you want to obtain insider information, or information that might give you an unfair advantage in the application process.

If you are not familiar with a specific required component, inform yourself before writing that element. A mistake to avoid is writing a statement about something you lack knowledge about. This lack of knowledge will be clear to the hiring committee. For example, for many academic positions in Canada, you will have to write a statement describing how you include equity, diversity, inclusion, and accessibility (EDIA) principles in your research and teaching. If you are unfamiliar with EDIA principles, then the best course of action is to find out about them in advance so that you can write an informed statement.

## Rule 3: Write a tailored job application

A common key mistake that applicants make is to submit a generic job application [17]. In fact, this rule is mentioned in most resources we have seen that provide advice on academic job applications [17–20]. Thus, it is crucial that you customize your application to show how your qualifications match the requirements of the specific position and that you will fit right in. To effectively do that, you first need to follow Rules 1 and 2. When reusing parts of previous application packages, double-check (or triple-check!) that details such as institution name, position title, and people’s names are correct. Receiving correspondence that seems meant for another institution or person leads to a rejection almost directly, as it is perceived as a lack of attention to detail.

When describing your qualifications and how you fit in, make sure that these descriptions are aligned with the job posting (see Rule 1). Use keywords and terminology used in the job ad. Committee members might not be experts in the hiring area and using other terms might raise questions about whether you meet the job requirements or simply generate confusion about your fit for the position. For example, if the job posting mentions that successful candidates must have expertise in ecology, then when describing your expertise, use the word ecology instead of synonyms or related terms such as environmental biology or bionomics.

Meizlish and Kaplan [21] found that search committees use teaching statements as evidence that a candidate has thought about teaching. Even if you do not have previous teaching experience, you can still show in your teaching documents that you have thought about how you will approach teaching by describing what type of teacher you envision yourself becoming. Thus, a good teaching statement should demonstrate a real commitment to teaching and serious reflection on one’s teaching, provide concrete evidence of teaching experience (if any), and avoid vague platitudes [10,21]. Like all application materials, it should be institution-specific. For example, one can mention what existing courses one might teach and what new courses one might introduce.

One might also need to modify one’s research plans to fit the institution; specifically, to avoid proposing research that cannot be carried out at a specific institution. There might be many reasons why specialized research might not be feasible at a given institution; for example, required research facilities or services might not exist, or, for certain life sciences, the institution might not have the required biosafety level.

## Rule 4: Identify others with caution

Identifying potential collaborators in your research statement shows that you have followed Rule 2. However, this can backfire if the research topics of the named potential collaborators are incorrect or obsolete. Applicants must be aware that information on web pages and on profiles in professional networks (e.g., ResearchGate, Google Scholar, and LinkedIn) might be out of date, that generative AI might hallucinate, and that research interests evolve. Our suggestion is to look at recent publications (within the past 6 years) of potential collaborators to confirm that they are still working on the identified research topic. Make sure to give more weight to publications coming from the potential collaborators’ labs to figure out their current areas of research. Identifying the “home lab” of publications might not be straightforward in multi-lab publications, as conventions for order of authorship vary across disciplines, countries, and even research groups. For instance, in many life sciences, authorship is in a V-shaped order of contribution, with the person who did most of the writing or the research being named first, and the senior author who contributed the most being listed last. In mathematics and computer science, order of authorship tends to be alphabetical. Thus, one should not rely on order of authorship to determine authors’ contributions; however, a good indicator of a particular lab’s involvement is the number of trainees from that lab listed as authors. Additionally, many journals require statements on author contributions. If such a statement is available, looking at it would be helpful to find out each author’s contribution. If you are uncertain whether a potential collaborator is still active in a given research area, we advise against naming them in your application without first reaching out to them to find out whether they are still active in the relevant area. This could also be an excellent opportunity to connect with potential collaborators.

## Rule 5: Set realistic expectations

Consider whether your teaching and research plans are feasible. In terms of teaching, even though it is good to show breadth in your teaching potential and interests, do not overdo it by claiming that you can teach any course, even if “given the appropriate amount of notice”. As for workload, check that time-wise your plans are doable; for example, you can be a good teacher without meeting weekly one-on-one with all your students to discuss their term project. Similarly, regarding research, it is good to describe novel ideas and a couple of specific projects to start in your own lab, but do not say that you plan to start a whole new research program outside your most evident area of expertise while applying for research grants and teaching new courses. Additionally, you might want to avoid proposing work for which there is no current infrastructure at the institution you are applying to. Alternatively, if that work is essential for your research program, be explicit about what facilities you would need to carry out the proposed research and, if possible, mention contingency plans or strategies you have identified to obtain the resources you will need. In sum, be realistic and avoid promising too much.

## Rule 6: Be careful with humorous or controversial assertions

Humor works best during in-person interviews or even in hiring seminars, where a personal connection can be more easily established with the audience. Avoid writing controversial, provocative, or humorous statements, as they might backfire or be misunderstood when put in written form. However, if you strongly feel that you must include such an assertion in writing, then provide context and justification so that reviewers have some background on how to interpret such a statement. We have encountered humorous or controversial assertions mostly in the teaching philosophy, EDIA statement, and cover letter, and for the most part, they seem out of place.

## Rule 7: Presentation matters

Hiring committee members go through tens (maybe hundreds) of application packages, so make yours as easy to read as possible. Start by choosing accessible fonts, uncluttered and visually engaging document layouts, colourblind-friendly palettes, and indexed sections for long documents. Make sure important information about you stands out while less important information does not take an overly prominent place. Some applicants emphasize key sentences using boldface or underlining. This is effective as long as it is used sparingly. However, we caution against using coloured fonts, highlighting, or shading. These can be distracting and reduce readability. Aim for a precise, concise, and clear writing style and do not forget to check spelling and grammar. Consider including well-designed and clear figures, particularly in your research statement to effectively communicate plans, methods, and results (see [22,23] for guidelines on how to create effective scientific figures).

## Rule 8: Confirm the willingness and availability of your referees to provide a letter of recommendation

Unfortunately, it is not a rare occurrence that referees will fail (or in rare cases refuse to) provide a letter of recommendation. To minimize this risk, ask your referees in advance and provide them with the information they need to write you a recommendation letter. Knapen et al. [24] provide excellent advice regarding obtaining positive recommendation letters: 1) select people who know you well and are willing to write detailed recommendation letters, 2) have a pool of referees and interchange them for different applications, and 3) if there are special circumstances with respect to the selection of referees (e.g., none of your direct supervisors is one of your referees), mention this in your application (e.g., make the committee aware why a potentially important referee is unavailable) or try to ask one of your other referees to address a special situation in terms which are positive for you. Usually, referees are expected to be academics, but there might be situations where including a nonacademic referee might be beneficial. For example, if the position has an industry component, such as establishing industry collaborations or carrying out industry-specific applied research, a nonacademic referee who can speak about your industry connections or industry applications of your research could be valuable. At least one of your references must be academic. Usually, in our experience, providing more references than the number requested in the job ad will not provide an advantage, as only the requested number of referees will be contacted. You might establish a priority list of references by putting the potentially most relevant persons first, and having those who might be least appropriate last, then selecting a number of them according to the number of requested references in the ad. This list might be modified from one ad to another, depending on what each institution is looking for.

## Rule 9: Carefully follow submission instructions

Hiring committees aim to have a systematic review of candidates, and thus set up guidelines for the candidates to follow. These guidelines might include, among others, the description of documents to include, page limits, file formats, file names, and procedures to obtain letters of reference. In some cases, the first filtering out of applicants will be made based on whether the candidates followed the basic guidelines. Thus, by not following the instructions provided, one risks that one’s application might not even reach the hiring committee. Furthermore, following submission instructions is a courtesy towards the hiring committee and not doing so will be noted in a negative way.

## Rule 10: Be authentic

Academic hiring committees are looking not only for good researchers and teachers, but also for good colleagues and team members. Candidates have to showcase and highlight their qualifications for and interest in the position, while at the same time avoiding coming across as bragging, exaggerating, or misleading. As it has been pointed out elsewhere [25], this is a fine line to walk. We suggest using adjectives and adverbs sparingly so that they are more effective when used. Additionally, we find that actually illustrating the significance, novelty, and impact of one’s research and contributions is more effective than piling up adjectives. It is more impressive to read a clear explanation of why something is novel, transformative and/or ground-breaking than to read a list of unsupported adjectives. For example, a phrase such as “My research strongly applies to the field of Wildlife Conservation” should be followed by good evidence supporting the statement. We also caution against using trite phrases/words such as “game-changing” as they might be perceived as lacking originality, or as being bombastic or boastful, especially if they are not followed by evidence proving the claim.

Additionally, as part of being authentic, your application documents should be written using your own words. AI tools based on large language models (LLMs), such as ChatGPT, Claude, or Gemini, are powerful tools that can help improve writing quality and correct grammar and spelling mistakes. However, LLMs are also designed to model the probabilistic distribution of human language, so their output is hegemonically average [26], intrinsically prone to utter false statements [27], and likely to produce hyperbole. For the experienced reader, AI-generated text is remarkably easy to spot. Therefore, we caution against using LLMs to write your job application documents from scratch. For ideas on how to effectively use these tools to assist you on writing job applications, please refer to [24].

## Conclusion

The academic job market is very competitive, and submitting a good job application package might increase your opportunities to land your desired job. Hiring committees are looking for good researchers, teachers, and colleagues with the best potential to match the job requirements. Successful candidates are those who express their ideas about teaching and research in an articulate way, and can clearly demonstrate in writing how they meet (or exceed) the selection criteria. We have delineated these rules as a guide for early-career academics to help them prepare a good job application, avoid critical mistakes, and be more likely to progress to an interview.
